# Supplementary material for: The Futile Creatine Cycle powers UCP1-independent thermogenesis in classical BAT
Source: Nat Commun. 2025 Apr 4;16:3221. doi: 10.1038/s41467-025-58294-4 (PMC11971250; doi:10.1038/s41467-025-58294-4)
Supplement: Supplementary file 1 — Supplementary Information [file 41467_2025_58294_MOESM1_ESM.docx]

Supplementary Information for

The Futile Creatine Cycle powers UCP1-independent thermogenesis in classical BAT

Jakub Bunk^1,2^, Mohammed F. Hussain^1,2,^, Maria Delgado-Martin^1,2,^, Bozena Samborska^1^, Mina Ersin^1,2^, Abhirup Shaw^1^, Janane F. Rahbani^1^, and Lawrence Kazak^1,2,^*.

^1^Rosalind & Morris Goodman Cancer Institute, McGill University, Montreal, QC, H3A 1A3, Canada

^2^Department of Biochemistry, McGill University, Montreal, QC, H3G 1Y6, Canada

Corresponding author: [lawrence.kazak@mcgill.ca](mailto:lawrence.kazak@mcgill.caxxxx.xxx)

**The PDF file includes:**

Supplementary Figs. 1 to 6


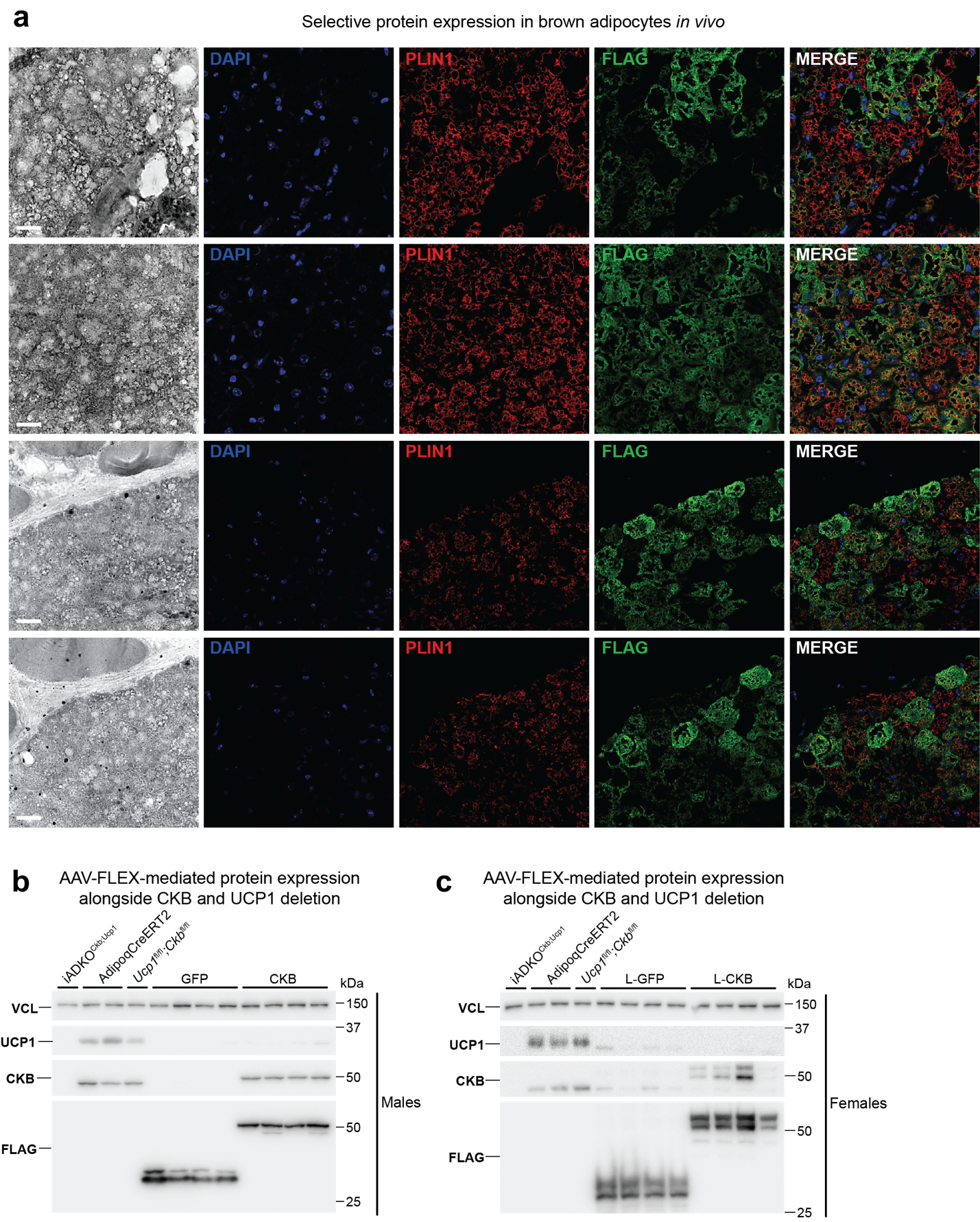


**Supplementary** **Fig. 1 | Simultaneous overexpression from AAV-FLEX in brown adipocytes and deletion of endogenous CKB and UCP1 in iBAT. a,** Representative immunofluorescence images of iBAT from female AdipoqCre^+^ mice transduced with AAV-FLEX-GFP-FLAG. Mature adipocytes were labelled with anti-Perilipin 1 (PLIN1) antibody (red), GFP-FLAG was labeled with anti-FLAG antibody (green). Nuclei were labelled with DAPI (blue). Scale bars, 20 μm. **b,** Western blot of iBAT harvested from 6- to 8-week-old iADKO^Ckb;Ucp1^, AdipoqCreERT2, and *Ucp1*^fl/fl^;*Ckb*^fl/fl^ male mice. iADKO^Ckb;Ucp1^ mice were injected with AAV-FLEX-GFP-FLAG (GFP) or AAV-FLEX-CKB-FLAG (CKB) subcutaneously above the iBAT (*n* = 4 per group). **c,** Western blot of iBAT harvested from 6- to 8-week-old iADKO^Ckb;Ucp1^, AdipoqCreERT2, and *Ucp1*^fl/fl^;*Ckb*^fl/fl^ female mice. iADKO mice were injected with AAV-FLEX-LACTB-GFP-FLAG (L-GFP) or AAV-FLEX-LACTB-CKB-FLAG (L-CKB) subcutaneously above the iBAT (*n* = 4 per group). *Ucp1*^fl/fl^;*Ckb*^fl/fl^ and AdipoqCreERT2 mice were used as controls for tamoxifen-inducible deletion of endogenous UCP1 and CKB. *n* numbers are of biologically independent experiments. Source data are provided as a Source Data file.


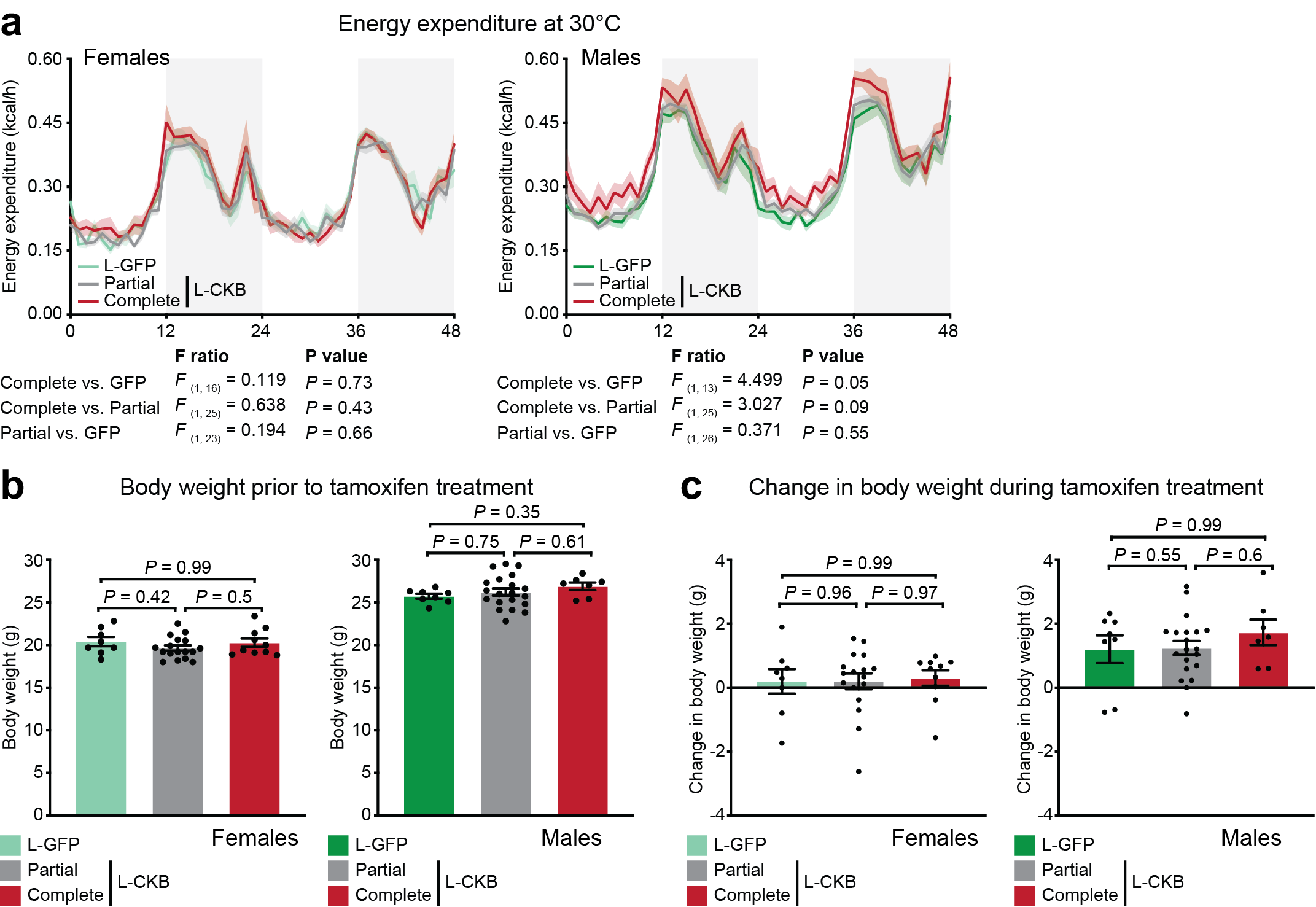


**Supplementary Fig. 2 | Energy expenditure and body weight of mice expressing L-GFP and L-CKB. a,** Energy expenditure (EE) at 30°C of 10- to 13-week-old iADKO^Ckb;Ucp1^ female or male mice expressing L-GFP (*n* = 8, 8), partial L-CKB (*n* = 17, 20), or complete L-CKB (*n* = 10, 7). **b,** Body weight of 8- to 11-week-old iADKO female or male mice (prior to tamoxifen treatment) expressing L-GFP (*n* = 8, 8), partial L-CKB (*n* = 17, 20), or complete L-CKB (*n* = 10, 7). **c,** Change in body weight between tamoxifen treatment and start of cold exposure of mice shown in Supplementary Fig. 2b. Data are presented as mean ± s.e.m. and *n* numbers are of biologically independent experiments. **a,** two-way ANOVA (Šidák post-hoc test); **b, c,** one-way ANOVA (Tukey’s post-hoc test). Source data are provided as a Source Data file.


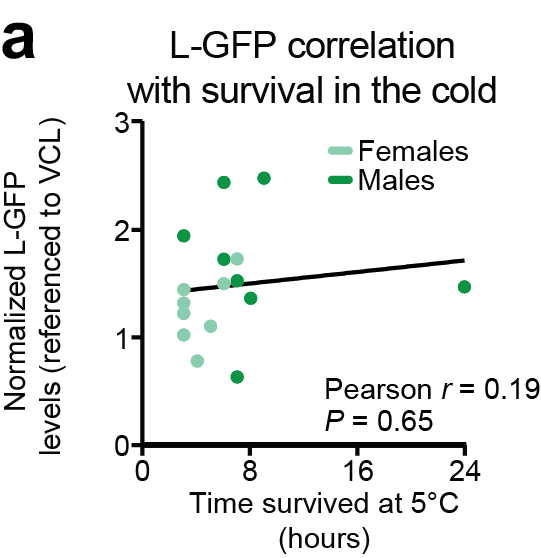


**Supplementary Fig. 3 | Correlation of L-GFP expression with survival in the cold. a,** Pearson correlation of L-GFP protein expression and survival time at 5°C (*n* = 8 per sex). *n* numbers are of biologically independent experiments. **a,** Pearson correlation (two-sided). Source data are provided as a Source Data file.


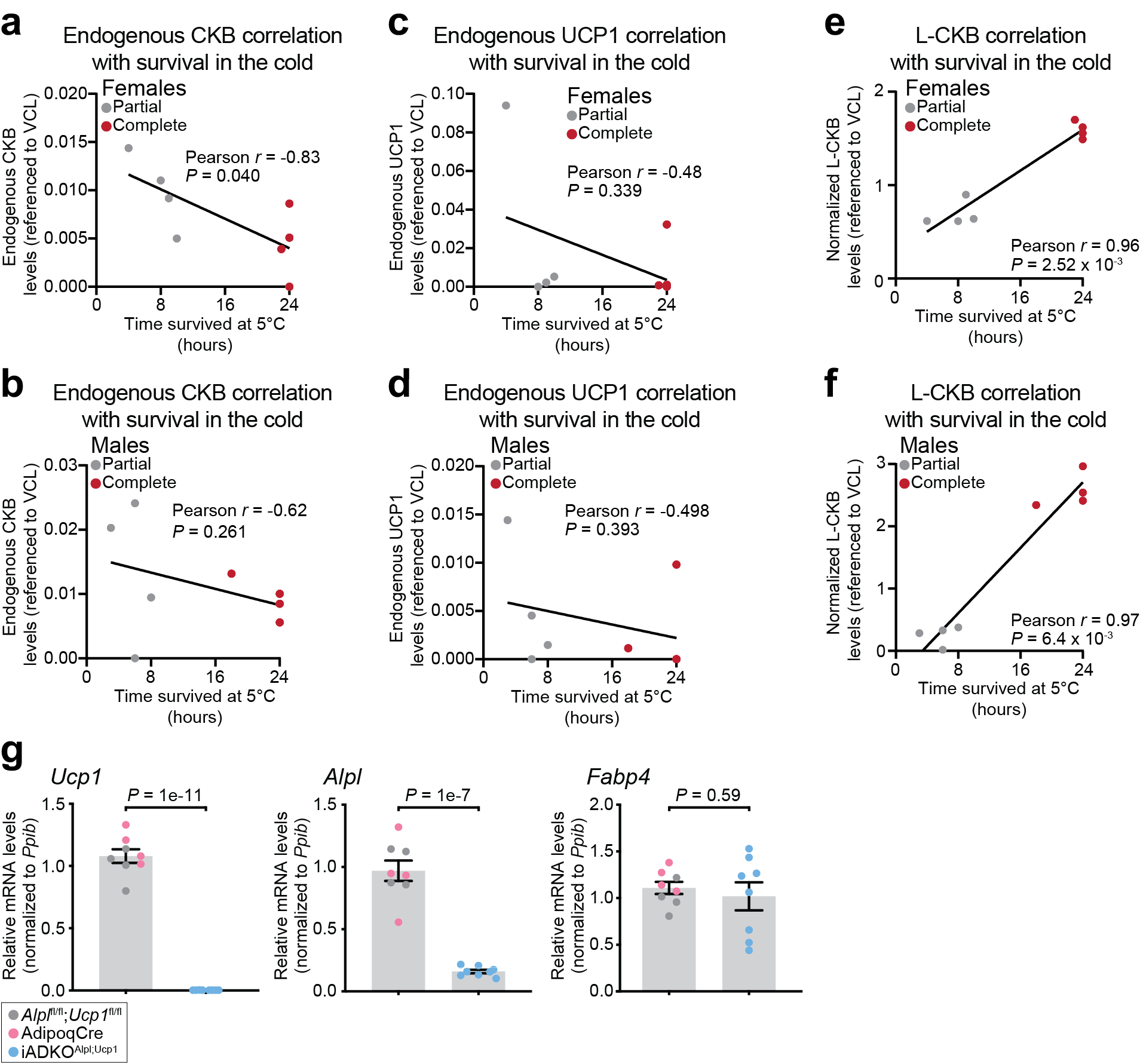
**Supplementary Fig. 4 | Correlation of endogenous CKB and UCP1, and L-CKB expression, with survival in the cold. a-b,** Pearson correlation of endogenous CKB protein expression and survival time at 5°C in (**a**) female and (**b**) male iADKO^Ckb;Ucp1^ mice (*n* = 8 per sex). **c-d,** Pearson correlation of endogenous UCP1 protein expression and survival time at 5°C in (**c**) female and (**d**) male iADKO mice (*n* = 8 per sex). **e-f,** Pearson correlation of L-CKB protein expression and survival time at 5°C in (**e**) female and (**f**) male iADKO^Ckb;Ucp1^ mice (*n* = 8 per sex). These correlations were obtained from the western blot in Fig. 4f. **g,** RT-qPCR from brown adipose tissue (BAT) of 10- to 11-week-old iADKO^Alpl;Ucp1^, AdipoqCreERT2, and *Alpl*^fl/fl^;*Ucp1*^fl/fl^ male mice (*n* = 8, 4, 4 per group). Data are presented as mean ± s.e.m. and *n* numbers are of biologically independent experiments. **a-f,** Pearson correlation (two-sided); **g,** two-tailed student’s t-test. Source data are provided as a Source Data file.


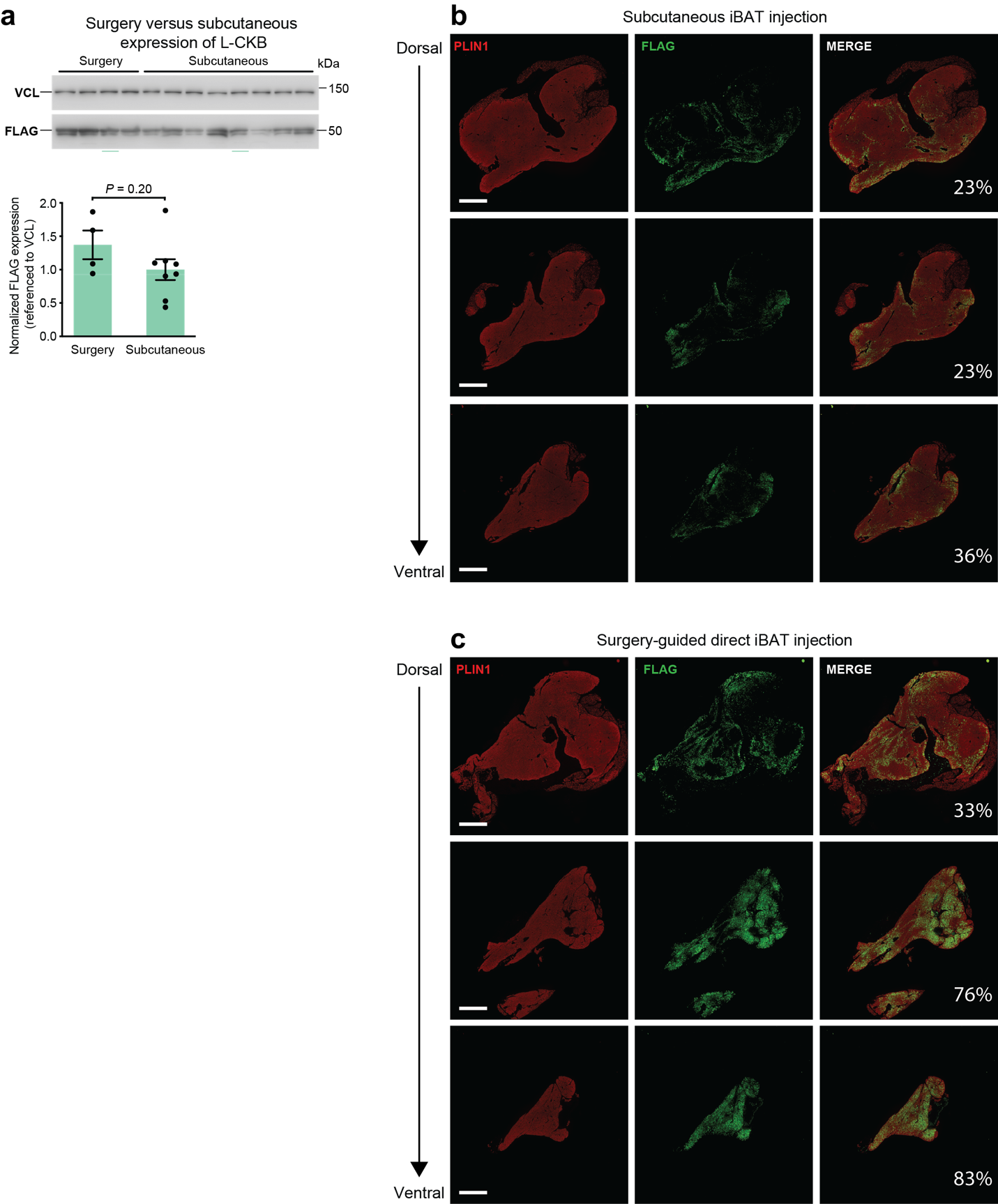


**Supplementary Fig. 5 | Efficiency of L-CKB protein expression from subcutaneous or surgically-guided AAV-FLEX-LACTB-CKB-FLAG** **transduction of iBAT. a**, Western blot of iBAT harvested from 9- to 11-week-old iADKO^Ckb;Ucp1^ male mice transduced with AAV-FLEX-LACTB-CKB-FLAG via subcutaneous or surgically-guided injection (*n* = 4 and 8 per group respectively). Quantification of relative L-CKB expression (referenced to VCL) from the western blot above. **b-c,** Immunofluorescence images of a serially sectioned iBAT lobe from one male iADKO^Ckb;Ucp1^ mouse transduced with AAV-FLEX-LACTB-CKB-FLAG via (**b**) subcutaneous or one male iADKO^Ckb;Ucp1^ mouse transduced with AAV-FLEX-LACTB-CKB-FLAG via (**c**) surgically-guided direct iBAT injection. Three different tissue depths are shown. The green line under the sample indicates the samples used for immunofluorescence. These were chosen because they exhibited similarly quantified expression levels by western blot, which allowed us to specifically gauge qualitatively if transduction could be as good with direct injections. Mature adipocytes were labelled with anti-Perilipin 1 (PLIN1) antibody (red), L-CKB was labeled with anti-FLAG antibody (green). Scale bars, 1,000 μm. Data are presented as mean ± s.e.m. and *n* numbers are of biologically independent experiments. **a,** two-tailed student’s t-test. Source data are provided as a Source Data file.


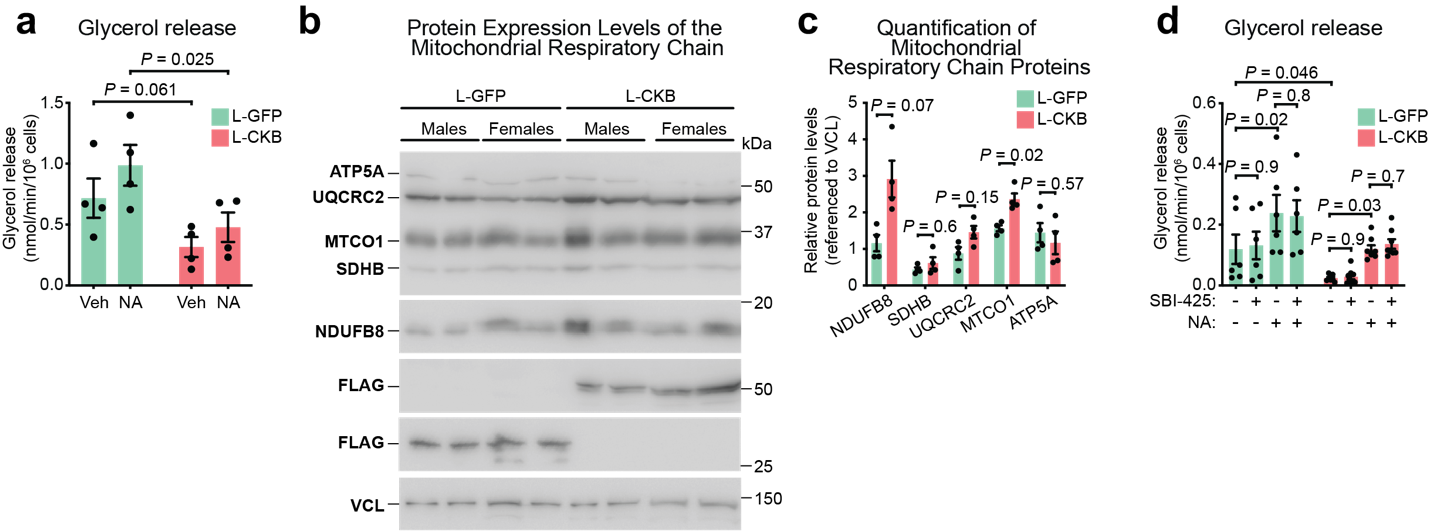
**Supplementary Fig. 6 | Lipolysis and respiratory chain abundance. a,** Glycerol release from brown adipocytes, acutely isolated from 12-week-old female and male iADKO^Ckb;Ucp1^ mice (*n* = 2 per sex per group). Vehicle, Veh; noradrenaline, NA. NA was used at 0.1 μM for 10 minutes. **b**, Western blot of brown adipocytes, acutely isolated from 12-week-old iADKO^Ckb;Ucp1^ female and male mice, (*n* = 2 per sex per group). **c,** Quantification of respiratory chain protein levels (referenced to VCL) from Supplementary Fig. 6b. **d**, Glycerol release from brown adipocytes, acutely isolated from 18-week-old iADKO^Ckb;Ucp1^ mice. SBI-425 (10 μM) and NA (0.1 μM) were used for 10 minutes. (*n* = 6 (2 females and 4 males for L-GFP); *n* = 8 (4 females and 4 males for L-CKB)). **a,** Two-way ANOVA (Tukey’s post-hoc test); **c,** Multiple unpaired *t*-test (Holm-Šídák correction); **d,** two-way ANOVA (Fisher’s least significant difference (LSD)). Source data are provided as a Source Data file.

Uncropped scans of blots and gels

**Supplementary Fig. 1b**

**
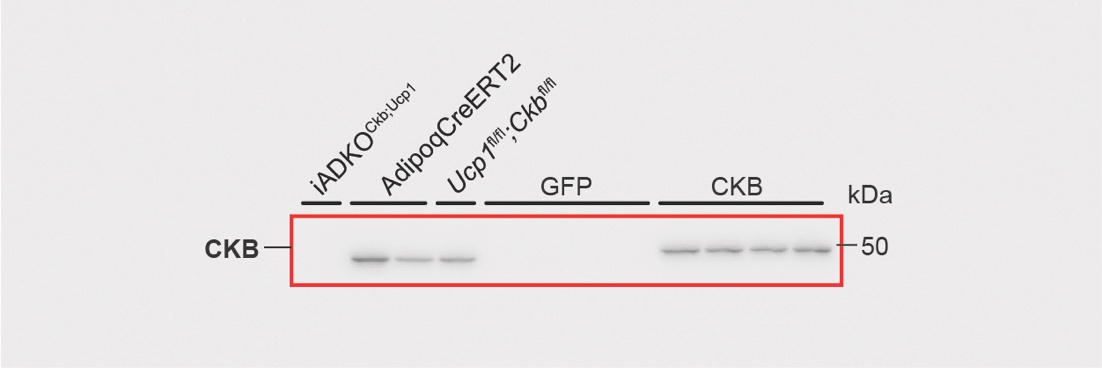
**
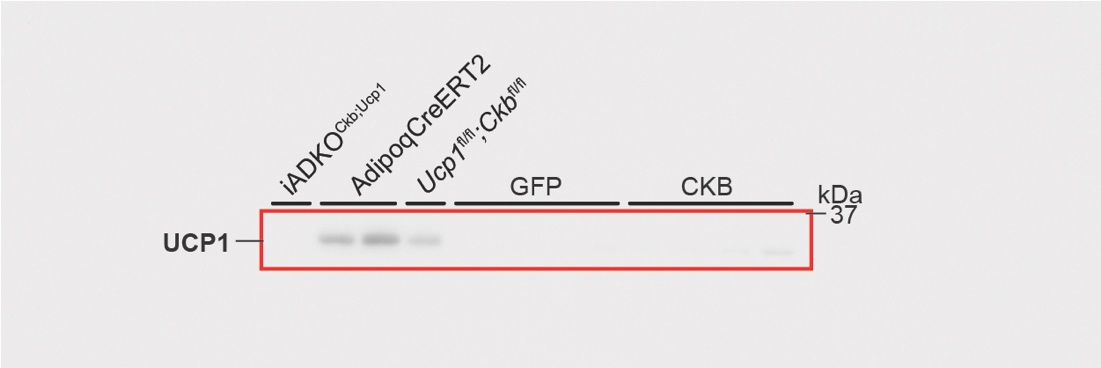

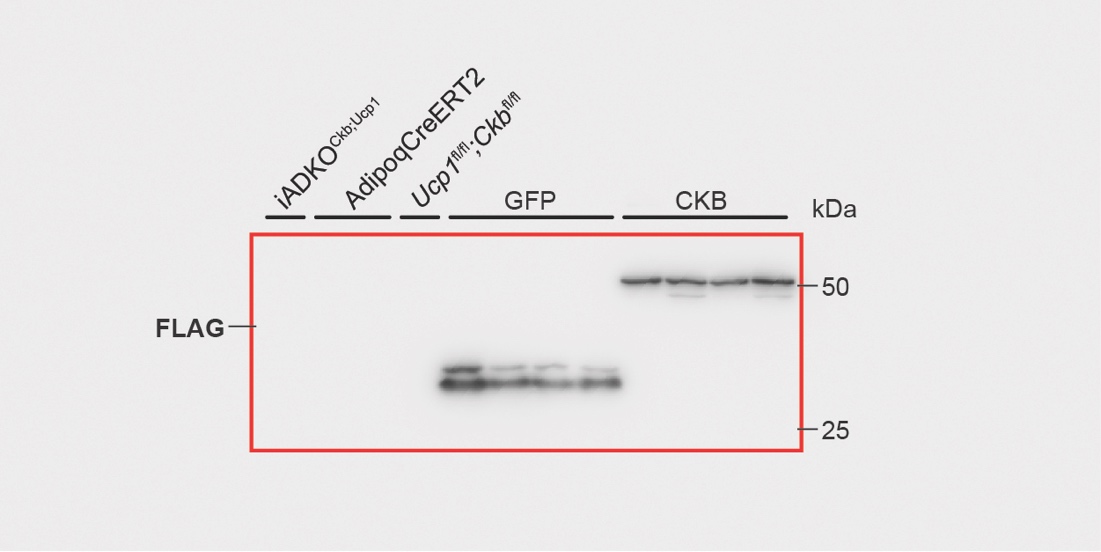


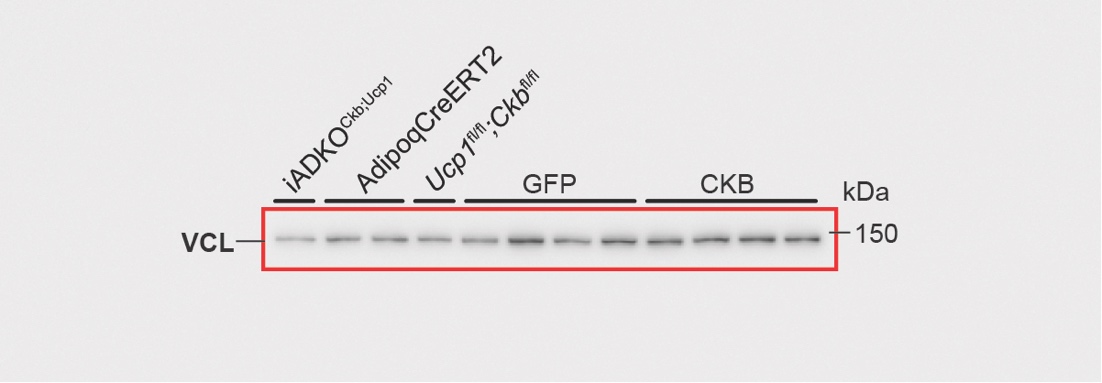

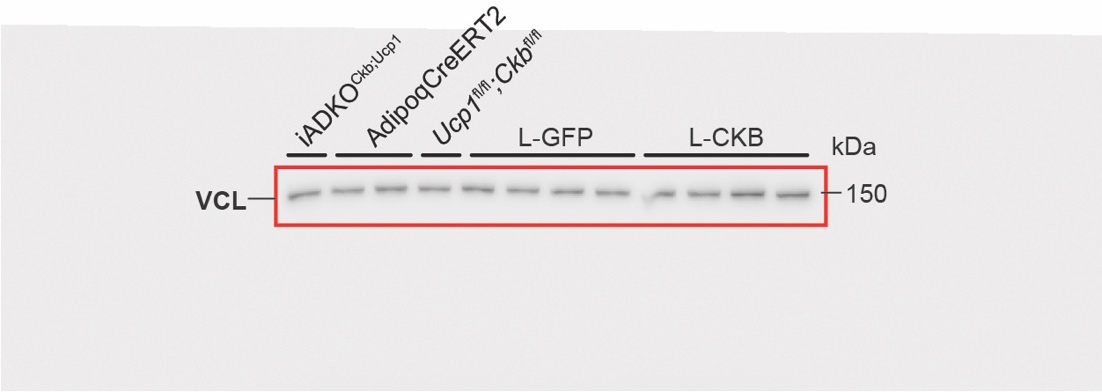

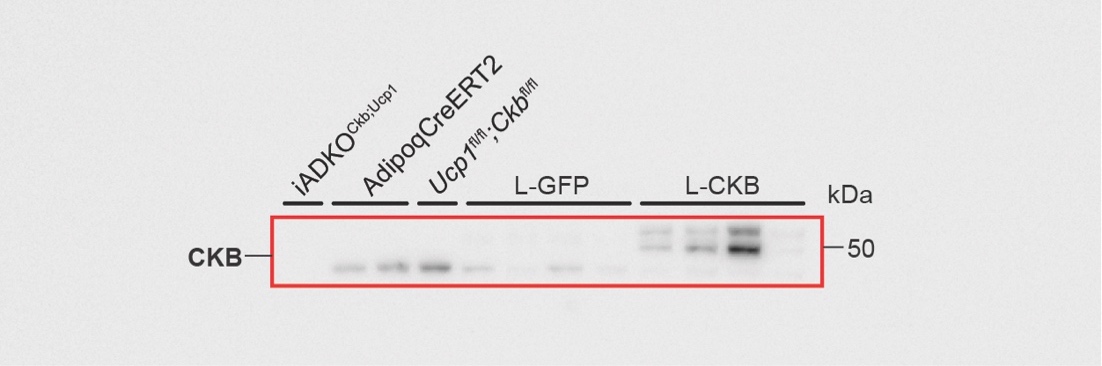

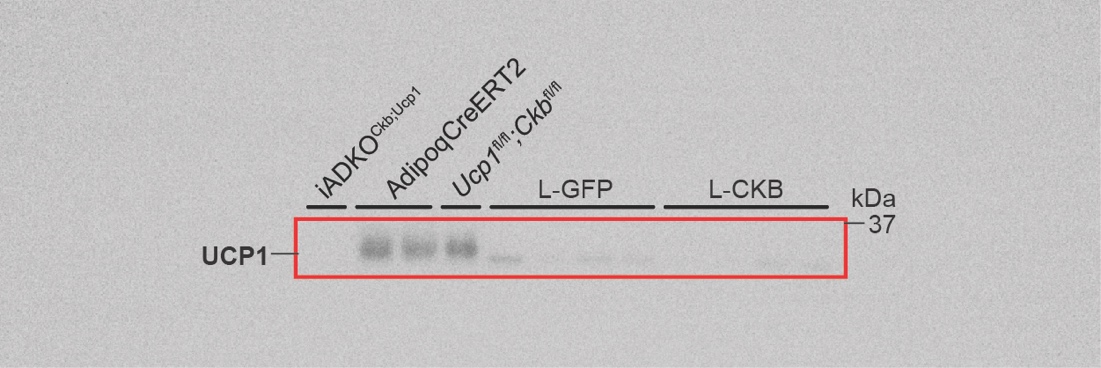

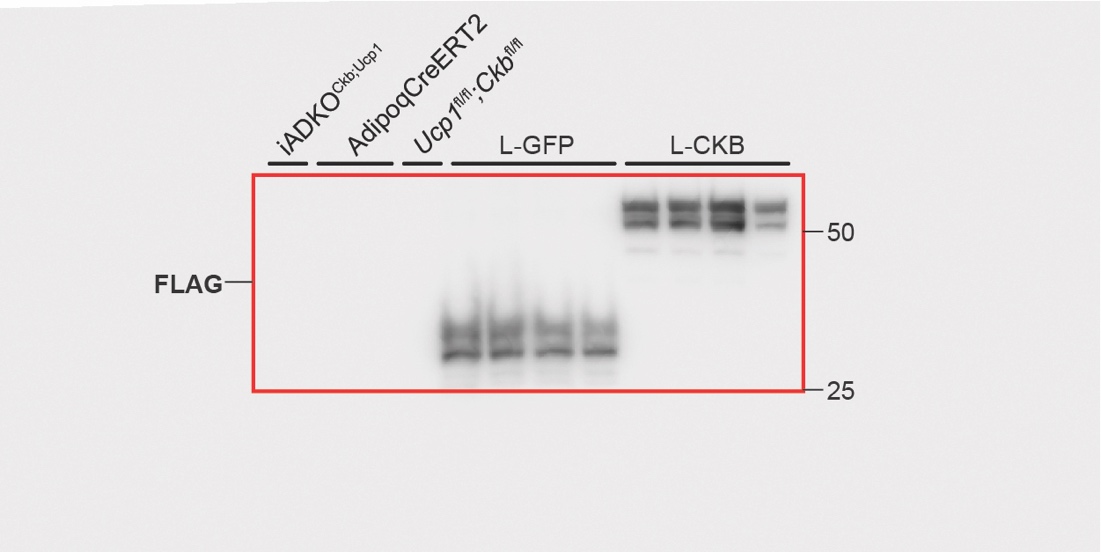
**Supplementary Fig. 1c**

**Supplementary Fig. 5a**


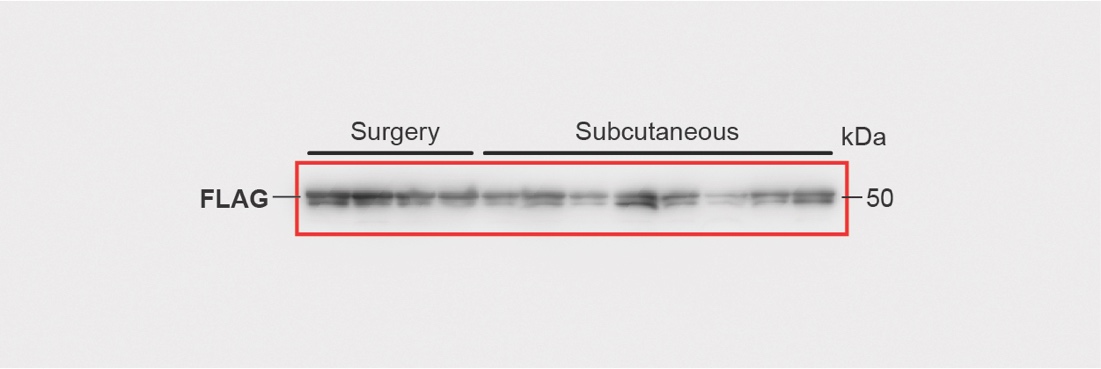

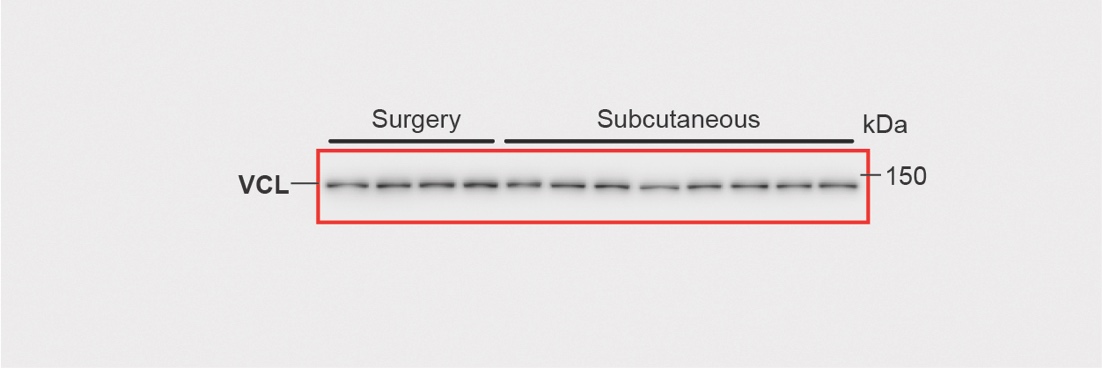


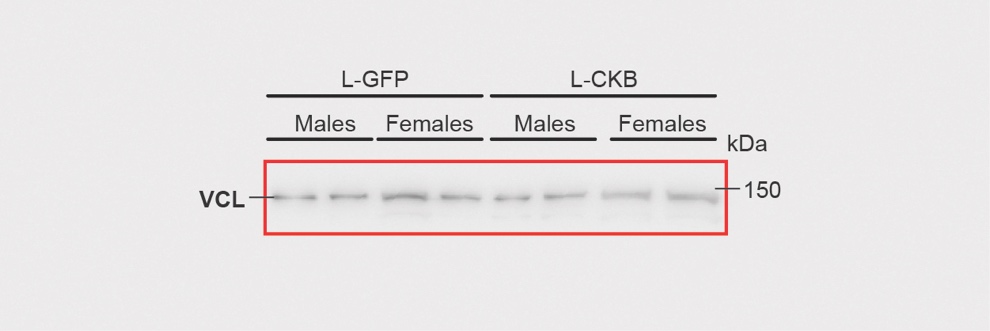
**
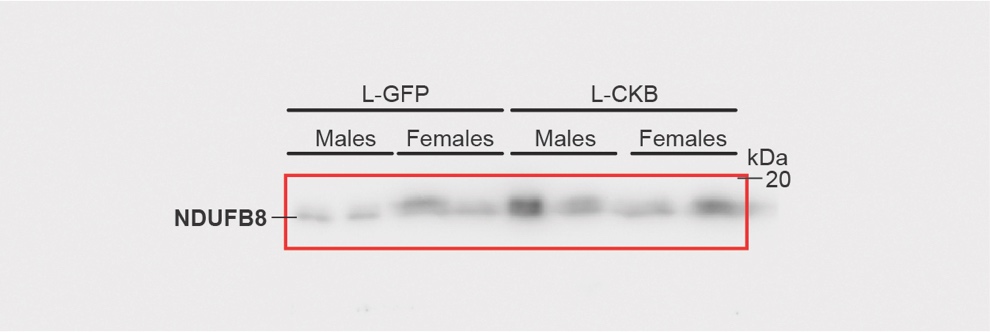

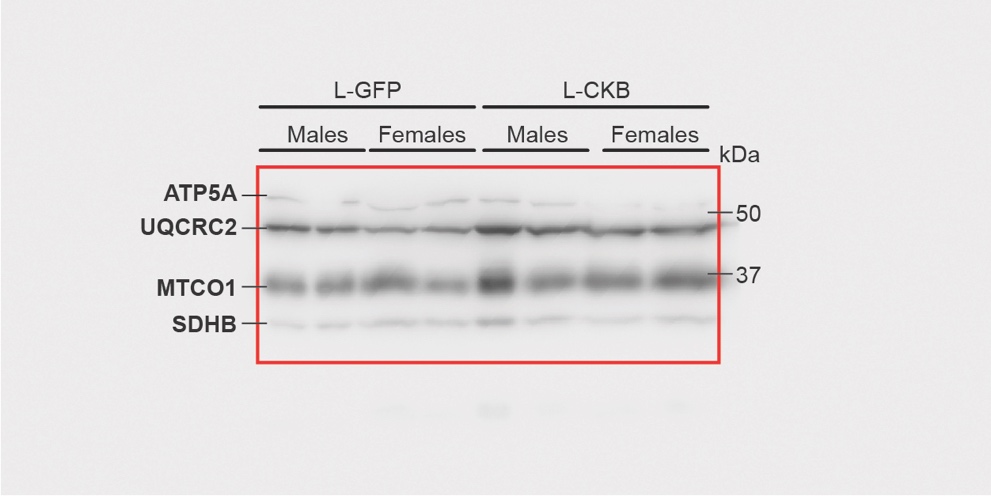
**
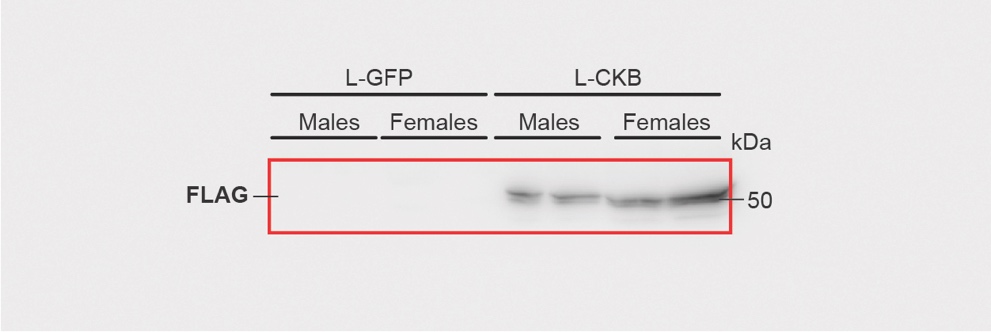

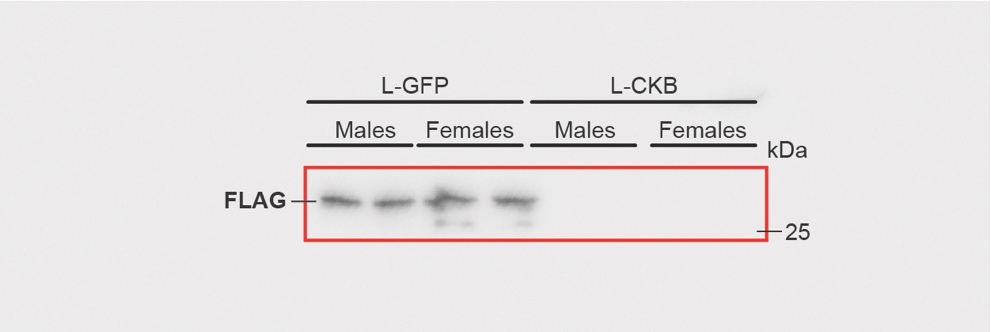
**Supplementary Fig. 6b**
