# Supplementary material for: The Futile Creatine Cycle powers UCP1-independent thermogenesis in classical BAT
Source: Nat Commun. 2025 Apr 4;16:3221. doi: 10.1038/s41467-025-58294-4 (PMC11971250; doi:10.1038/s41467-025-58294-4)
Supplement: Supplementary file 2 — Reporting Summary [file 41467_2025_58294_MOESM2_ESM.pdf]

nature portfolio

Corresponding author(s): Lawrence Kazak

Last updated by author(s): Mar 3, 2025

Reporting Summary

Nature Portfolio wishes to improve the reproducibility of the work that we publish. This form provides structure for consistency and transparency in reporting. For further information on Nature Portfolio policies, see our Editorial Policies and the Editorial Policy Checklist.

Statistics

For all statistical analyses, confirm that the following items are present in the figure legend, table legend, main text, or Methods section.

n/a	Confirmed	
[ ]	[x]	The exact sample size (*n*) for each experimental group/condition, given as a discrete number and unit of measurement
[ ]	[x]	A statement on whether measurements were taken from distinct samples or whether the same sample was measured repeatedly
[ ]	[x]	The statistical test(s) used AND whether they are one- or two-sided<br>*Only common tests should be described solely by name; describe more complex techniques in the Methods section.*
[x]	[ ]	A description of all covariates tested
[ ]	[x]	A description of any assumptions or corrections, such as tests of normality and adjustment for multiple comparisons
[ ]	[x]	A full description of the statistical parameters including central tendency (e.g. means) or other basic estimates (e.g. regression coefficient) AND variation (e.g. standard deviation) or associated estimates of uncertainty (e.g. confidence intervals)
[ ]	[x]	For null hypothesis testing, the test statistic (e.g. *F*, *t*, *r*) with confidence intervals, effect sizes, degrees of freedom and *P* value noted<br>*Give P values as exact values whenever suitable.*
[x]	[ ]	For Bayesian analysis, information on the choice of priors and Markov chain Monte Carlo settings
[x]	[ ]	For hierarchical and complex designs, identification of the appropriate level for tests and full reporting of outcomes
[ ]	[x]	Estimates of effect sizes (e.g. Cohen's *d*, Pearson's *r*), indicating how they were calculated

Our web collection on statistics for biologists contains articles on many of the points above.

Software and code

Policy information about availability of computer code

Data collection	Indirect calorimetry: Sable Systems International, Promethion high-definition behavioural phenotyping system data acquisition software (IM-3 v.23.0.4).<br>QPCR: CFX Maestro 2017<br>Western blotting: Bio-Rad Chemidoc Imaging System<br>Widefield epifluorescent microscopy: Zeiss Axio Observer inverted microscope, Axiocam 506m CCD camera (Zeiss)<br>STED super-resolution microscopy: inverted Stellaris-8 confocal microscope (Leica), HyD X detector, HyD S detector
Data analysis	Systems International MacroInterpreter software (v.23.6.0) using One-Click Macro (v.2.51.0).<br>GraphPad Prism, 9<br>Microsoft office Excel (v.16.77.1)<br>ImageJ (v.1.51s)<br>Zen Blue software (v.2.6)<br>Leica Application Suite X (v.4.6.1.27508)

For manuscripts utilizing custom algorithms or software that are central to the research but not yet described in published literature, software must be made available to editors and reviewers. We strongly encourage code deposition in a community repository (e.g. GitHub). See the Nature Portfolio guidelines for submitting code & software for further information.

Data

Policy information about availability of data

All manuscripts must include a data availability statement. This statement should provide the following information, where applicable:

- Accession codes, unique identifiers, or web links for publicly available datasets
- A description of any restrictions on data availability
- For clinical datasets or third party data, please ensure that the statement adheres to our policy

Source data are provided with this paper

Research involving human participants, their data, or biological material

Policy information about studies with human participants or human data. See also policy information about sex, gender (identity/presentation), and sexual orientation and race, ethnicity and racism.

Reporting on sex and gender	N/A
Reporting on race, ethnicity, or other socially relevant groupings	N/A
Population characteristics	N/A
Recruitment	N/A
Ethics oversight	N/A

Note that full information on the approval of the study protocol must also be provided in the manuscript.

Field-specific reporting

Please select the one below that is the best fit for your research. If you are not sure, read the appropriate sections before making your selection.

☒ Life sciences ☐ Behavioural & social sciences ☐ Ecological, evolutionary & environmental sciences

For a reference copy of the document with all sections, see nature.com/documents/nr-reporting-summary-flat.pdf

Life sciences study design

All studies must disclose on these points even when the disclosure is negative.

Sample size	Sample sizes were predetermined based on effect size, standard deviation, and significance level required to attain statistical significance of p<0.05 with a 90% probability on the basis of previous experiments using similar methodologies and were deemed sufficient to account for any biological/technical variability (PMIDs: 31161155, 30078553, 28844881, 24439384). For experiments without predetermination, sample sizes were chosen on the basis of prior experience and published standards in the field (see PMIDs above). Sample sizes are indicated for each experiment in the manuscript.
Data exclusions	No data were excluded
Replication	All attempts of replication were successful. Sample sizes for each experiment are reported in the figure legends. Western blots represent independent biological samples.
Randomization	For in vivo studies, mice in each genotype were randomly assigned to treatment groups.
Blinding	Experimenters were not blinded to experimental conditions, because data collection on indirect calorimetry is automated and confers high objectivity.

Reporting for specific materials, systems and methods

We require information from authors about some types of materials, experimental systems and methods used in many studies. Here, indicate whether each material, system or method listed is relevant to your study. If you are not sure if a list item applies to your research, read the appropriate section before selecting a response.

Materials & experimental systems

n/a	Involved in the study
☐	☒ Antibodies
☐	☒ Eukaryotic cell lines
☒	☐ Palaeontology and archaeology
☐	☒ Animals and other organisms
☒	☐ Clinical data
☒	☐ Dual use research of concern
☒	☐ Plants

Methods

n/a	Involved in the study
☒	☐ ChIP-seq
☒	☐ Flow cytometry
☒	☐ MRI-based neuroimaging

Antibodies

Antibodies used	VCL (Cell Signaling; cat. no. 13901; clone E1E9V): diluted at 1:5,000<br>CKB (Abclonal; cat. no. ab12631): diluted at 1:1,000<br>CKB (Abcam; cat. no. ab125114): diluted at 1:200<br>UCP1 (Abcam; cat. no. ab10983): diluted at 1:2,000<br>TNAP 549 (R&D; cat. no. AF2910): diluted at 1:200<br>TH (Millipore Sigma; cat. no. ab152): diluted at 1:1,000<br>Total oxphos (Abcam; cat. no. ab110413): diluted at 1:10,000<br>HSP60 (Abcam; cat. no. ab46798): diluted at 1:10,000<br>LONP1 (Abcam; cat. no. ab103809): diluted at 1:1,500<br>Anti-rabbit (Promega; cat. no. W401B): diluted at 1:10,000 (v/v) in TBS-T containing 5% milk<br>Anti-mouse (Promega; cat. no. W402B): diluted at 1:10,000 (v/v) in TBS-T containing 5% milk
Validation	All antibodies are commercial in origin. Validation statements can be found on the manufacturer's website for the following:<br><br>VCL (Cell Signaling; Cat. No. 13901; clone E1E9V): https://www.cellsignal.com/products/primary-antibodies/vinculin-e1e9v-xp-rabbit-mab/13901<br>CKB (Abclonal; Cat. No. ab1263): https://abclonal.com/catalog-antibodies/CKBPolyclonalAntibody/A12631<br>CKB (Abcam; cat. no. ab125114): https://www.abcam.com/products/primary-antibodies/creatine-kinase-b-type-antibody-epr3927-ab92452.html<br>UCP1 (Abcam; Cat. No. ab10983): https://www.abcam.com/ucp1-antibody-ab10983.html<br>TNAP 549 (R&D; cat. no. AF2910): https://www.rndsystems.com/products/mouse-alkaline-phosphatase-alpl-antibody_af2910<br>TH (Millipore Sigma; cat. no. ab152): https://www.emdmillipore.com/CA/en/product/Anti-Tyrosine-Hydroxylase-Antibody,MM_NF-AB152?ReferrerURL=https%3A%2F%2Fwww.google.com%2F&bd=1<br>Total oxphos (Abcam; cat. no. ab110413): https://www.abcam.com/products/panels/total-oxphos-rodent-wb-antibody-cocktail-ab110413.html<br>HSP60 (Abcam; cat. no. ab46798): https://www.abcam.com/products/primary-antibodies/hsp60-antibody-ab46798.html<br>LONP1 (Abcam; cat. no. ab103809): https://www.abcam.com/products/primary-antibodies/lonp1lon-antibody-ab103809.html

Eukaryotic cell lines

Policy information about cell lines and Sex and Gender in Research

Cell line source(s)	HEK 293T cells
Authentication	HEK 293T cells were authenticated on the basis that they were able to generate functional AAVs
Mycoplasma contamination	Cell lines were not tested for mycoplasma.
Commonly misidentified lines (See ICLAC register)	No commonly misidentified cells were used in this study.

Animals and other research organisms

Policy information about studies involving animals; ARRIVE guidelines recommended for reporting animal research, and Sex and Gender in Research

Laboratory animals	iADKO mice have been previously described1. AdipoqCre+ and AdipoqCre- littermates were derived from breeding B6.FVB-Tg (Adipoq-cre)1Evdr/J (JAX stock #028020) with wild-type C57BL/6J (JAX stock #000664) mice. Mouse experiments were performed according to procedures approved by the Animal Resource Centre at McGill University and complied with guidelines set by the Canadian Council of Animal Care. The photoperiod was fixed at a 12-h light/ 12-h dark schedule (light 07:00 to 19:00) with lights on at 07:00 hours being defined as Zeitgeber time 0 (ZT0). Mice had ad libitum access to drinking water and a chow diet (3.1 kcal/g energy density) with 24%, 16%, and 60% of Calories from protein, fat, and carbohydrate, respectively (2920X, Envigo, Madison, WI, USA). All mice were born and housed in groups (3-5 mice per cage) at 23ºC 1ºC with bedding and shredded paper strips in the cage until experimental intervention (6-12 weeks of age). Suitable housing temperature of mice to optimally mimic human physiology is

	disputed. We followed the suggestion that when provided with bedding and nesting materials standard room temperature (22–24ºC) is appropriate50. For cold exposure experiments, mice were singly housed in cages with bedding and with ad libitum access to drinking water and chow diet. Mouse experiments used age-matched littermates and were conducted at the temperature indicated in each figure legend.
Wild animals	The study did not involve wild animals.
Reporting on sex	Findings apply to both sexes. Sex of mice used for experiments is noted in the Figure legends an in the source data.
Field-collected samples	The study did not involve samples collected from the field.
Ethics oversight	Animal experiments were performed according to procedures approved by the Animal Resource Centre at McGill University and complied with guidelines set by the Canadian Council of Animal Care.

Note that full information on the approval of the study protocol must also be provided in the manuscript.

Plants

Seed stocks	N/A
Novel plant genotypes	N/A
Authentication	N/A
